# Class I histone deacetylase inhibitor MS-275 attenuates vasoconstriction and inflammation in angiotensin II-induced hypertension

**DOI:** 10.1371/journal.pone.0213186

**Published:** 2019-03-04

**Authors:** Yuhee Ryu, Hae Jin Kee, Simei Sun, Young Mi Seok, Sin Young Choi, Gwi Ran Kim, Seung-Jung Kee, Marc Pflieger, Thomas Kurz, Hyung-Seok Kim, Myung Ho Jeong

**Affiliations:** 1 Heart Research Center of Chonnam National University Hospital, Gwangju, Republic of Korea; 2 Hypertension Heart Failure Research Center, Chonnam National University Hospital, Gwangju, Republic of Korea; 3 Molecular Medicine, Brain Korea 21 Plus, Chonnam National University Graduate School, Gwangju, Republic of Korea; 4 Zhoushan Hospital, Zhejiang University School of Medicine, Lincheng New District Zhoushan Zhejiang, China; 5 National Development Institute of Korean Medicine, Hwarang-ro, Gyeongsan-si, Gyeongsangbuk-do, Republic of Korea; 6 Department of Laboratory Medicine, Chonnam National University, Medical School and Hospital, Gwangju, Republic of Korea; 7 Institute of Pharmaceutical and Medicinal Chemistry, Heinrich Heine University Düsseldorf, Universitätsstr, 1, Düsseldorf, Germany; 8 Department of Forsensic Medicine, Chonnam National University Medical School, Gwangju, Republic of Korea; Max Delbruck Centrum fur Molekulare Medizin Berlin Buch, GERMANY

## Abstract

**Objective:**

Non-selective histone deacetylase (HDAC) inhibitors are known to improve hypertension. Here, we investigated the therapeutic effect and regulatory mechanism of the class I HDAC selective inhibitors, MS-275 and RGFP966, in angiotensin (Ang) II-induced hypertensive mice.

**Methods and results:**

MS-275 inhibited the activity of HDAC1, HDAC2, and HDAC3, while RGFP966 weakly inhibited that of HDAC3 in a cell-free system. MS-275 and RGFP966 treatment reduced systolic blood pressure and thickness of the aorta wall in Ang II-induced hypertensive mice. MS-275 treatment reduced aorta collagen deposition, as determined by Masson’s trichrome staining. MS-275 decreased the components of the renin angiotensin system and increased vascular relaxation of rat aortic rings via the nitric oxide (NO) pathway. NO levels reduced by Ang II were restored by MS-275 treatment in vascular smooth muscle cells (VSMCs). However, MS-275 dose (3 mg·kg^-1^·day^-1^) was not enough to induce NO production in vivo. In addition, MS-275 did not prevent endothelial nitric oxide synthase (eNOS) uncoupling in the aorta of Ang II-induced mice. Treatment with MS-275 failed to inhibit Ang II-induced expression of NADPH oxidase (Nox)1, Nox2, and p47phox. MS-275 treatment reduced proinflammatory cytokines such as tumor necrosis factor (TNF)-α, interleukin (IL)-1β, and monocyte chemoattractant protein (MCP)-1, as well as adhesion molecules. Histological analysis showed that Ang II-induced macrophage infiltration was reduced by MS-275 and RGFP966 administration.

**Conclusions:**

Our results indicate that class I HDAC selective inhibitors may be good therapeutic agents for the treatment of hypertension through the regulation of vascular remodeling and vasoconstriction, as well as inflammation.

## Introduction

Hypertension is a complex disease caused by genetic and environmental risk factors. It is one of the most important risk factors for cardiovascular disease and stroke events [1–4]. Numerous pathophysiological factors influence the development of hypertension. The increase in sodium intake, vascular stiffness, endothelial dysfunction, activated sympathetic nervous system (SNS), and renin-angiotensin-aldosterone system (RAAS) activation contributes to the pathogenesis of hypertension [5–7]. Although there are many effective antihypertensive therapies, managing hypertension is difficult in numerous patients. RAAS is the most studied mechanism of hypertension [8], and among its components, Ang II is a strong vasoconstrictor and elevator of blood pressure [9].

Moreover, Ang II is associated with oxidative stress and endothelial dysfunction [10]. The balance of endogenous vasoconstrictors and vasodilators plays a critical role in the homeostasis of vascular tone and vascular remodeling [11]. Endothelial dysfunction promotes high blood pressure. Nitric oxide (NO) is a gaseous vasodilator that acts as a protective mediator in the development of atherosclerosis [12]. Physiologically, NO plays a key role in the vasculature. However, under pathological states, endothelial NO synthase (eNOS) produces superoxide instead of NO because of eNOS uncoupling [13, 14]. Tetrahydrobiopterin (BH4) is a very important cofactor of eNOS activity and function [15, 16].

Hypertension is associated with the production of superoxide, formed by several oxidases and oxygenases such as NADPH oxidases (Nox), vascular peroxidase 1 (VPO1), and cyclooxygenase-2 (Cox)-2 [17]. Superoxide generated by Nox is metabolized by superoxide dismutase (SOD) to form hydrogen peroxide (H_2_O_2_) [18, 19].

Gene expression can be regulated by histone modifications. Among them, acetylation and deacetylation are modulated by histone acetylase (HAT) and histone deacetylase (HDAC), respectively. The expression and activity of various HDACs may be changed in diseases. HDAC inhibitors have been extensively studied in the field of cancer [20]. HDAC inhibitors have been studied in cardiovascular diseases including cardiac hypertrophy [21]. Cardinale et al. [22] first reported that long-term treatment with the pan-HDAC inhibitor valproic acid (VPA) reduces cardiac hypertrophy, inflammation, and hypertensive responses in spontaneously hypertensive rats (SHR). Recently, it was reported that HDAC3 and HDAC4 mediate hypertension such as in deoxycorticosterone acetate (DOCA)-salt-induced hypertensive rat and SHR, respectively [22]. The class I HDAC inhibitor, MS-275, attenuates hypertension and hyperglycemia in a model of Cushing’s syndrome [23]. A more recent study showed that VPA prevents high-fat diet-induced hypertension by downregulating Ang II and its receptor, AT1 [24]. Moreover, the pan-HDAC inhibitor trichostatin A (TSA) inhibits hypertension and vasoconstriction through AT1 [25]. Our previous study showed that MC1568, an HDAC inhibitor, reduces high systolic blood pressure and HDAC4 phosphorylation is increased in the kidney and thoracic aorta of Ang II-induced hypertensive mice [26]. Although cardiac HDAC6 activity was shown to be increased in chronic hypertension [27], the HDAC6-selective inhibitor tubastatin A did not reduce hypertension in Ang II-infused mice [28]. Recently, we reported that the protein levels of class IIa/b HDACs (HDAC4,5,7, 6, and 10) are induced in SHR hearts [29] but not in Ang II mouse hearts. Currently, the HDAC isoform that likely plays a key role in the regulation of hypertension remains unclear. Therefore, we investigated whether class I HDACs are involved in the regulation of hypertension. In this study, we evaluated the effect of MS-275 and RGFP966 on hypertension induced by Ang II infusion in mice. MS-275 is known to inhibit class I HDACs, whereas RGFP966 is a well-known inhibitor of HDAC3. However, there is misleading evidence about the HDAC inhibitors. To confirm the HDAC enzyme activities of MS-275 and RGFP966, we assessed HDAC activity in a cell-free system. To explore the mechanism by which HDAC inhibitors act, we investigated the RAAS, vascular contraction-relaxation response, arterial remodeling, oxidative stress, and inflammation in vivo.

## Materials and methods

### Animal model and blood pressure measurement

All animal experiments were approved by the Animal Experiment Committee of the Chonnam National University Medical School (CNU IACUC-H-2017-70) and carried out in accordance with the Guide for the Care and Use of Laboratory Animals (US National Institutes of Health Publication, 8^th^ edition, 2011). CD-1 male mice (8-week-old) were purchased from OrientBio Company (Gyeonggi, South Korea). Alzet mini-osmotic pumps (model 1002) were purchased from Durect Corporation (Cupertino, CA, USA).

Mice were anesthetized with a ketamine (120 mg/kg), and xylazine (6.2 mg/kg) mixture and 1-cm incisions were created on the backs of the mice. Ang II (1.3 mg·kg^-1^·day^-1^) was subcutaneously infused into the mice using an Alzet osmotic pump as described previously [26]. Mice were divided into four groups (n = 8 each): vehicle-treated controls, Ang II-infused, Ang II-infused with MS-275 treatment (Ang II + MS-275), and Ang-II-infused with RGFP966 treatment (Ang II + RGFP966). Ang II was dissolved in a 0.9% sodium chloride (NaCl) solution. The sham control mice received the vehicle (dimethyl sulfoxide [DMSO]). MS-275 and RGFP966 (both 3 mg·kg^-1^·day^-1^) were intraperitoneally injected into mice from day 8 to 14 after Ang II infusion. Mice were subjected to blood pressure measurements three times before determination of the systolic blood pressure on day 14 after Ang II infusion. The blood pressure was measured in awake mice using the tail-cuff method (BP-2000, Visitech Systems, Apex, NC, USA). Mice were sacrificed at the end of the study by CO_2_ inhalation.

### Fluorogenic class I HDAC enzyme activities

To evaluate the HDAC enzyme inhibitory activity of MS-275 and RGFP966, we determined enzyme activities of HDAC1, HDAC2, HDAC3, and HDAC8 using enzyme assay kits (BPS Bioscience, San Diego, CA, USA) according to the manufacturer’s protocols. TSA was evaluated as a reference compound. HDAC enzyme activities were measured using a fluorometer (Spectra Max GEMINI XPS, Molecular Devices, Sunnyvale, CA, USA) at excitation and emission wavelengths of 350 nm and 460 nm, respectively. To test the enzyme activity of MS-275, RGFP966, and TSA, 0.001, 0.003, 0.01, 0.03, 0.1, 0.3, 1, 3, and 10 μM concentrations were used. For the half-maximal inhibitory concentration (IC_50_) calculations, every data point was normalized to the vehicle (100% activity). The normalized data were fitted using a Hill nonlinear curve fit (OrigionPro 9.0). The “Find X from Y” function in OrigionPro 9.0 was used to determine the IC_50_ values (50% activity).

### Histology and immunohistochemical staining

Aortic tissues were fixed in 4% paraformaldehyde at 25°C, embedded in paraffin, and cut into 3-μm thick sections. The tissue slides were deparaffinized three times with xylene and hydrated using serially diluted ethanol. After immersing in tap water for 2 min, the slides were stained with Gill’s hematoxylin V for 5 min and then washed with tap water for 5 min and 95% ethanol for 2 min. The slides were stained with Eosin Y for 1 min, dehydrated with ethanol and xylene, and mounted using Canada balsam. The aortic wall thickness was measured using the NIS Elements Software (Nikon, Japan). Masson’s trichrome staining and orcein staining were used to identify collagen fibers and elastic fibers, respectively.

For immunohistochemistry, staining was performed in aortic tissues using a primary CD68 antibody. Before incubation with primary antibodies, aorta sections received antigen retrieval using a citrate buffer. Negative controls were performed without a primary antibody.

### Isometric tension measurement

A vasoconstriction-relaxation study was performed as described previously [30]. Male Sprague-Dawley rats were purchased from Orient Bio (Gyeonggi-do, South Korea). Briefly, the thoracic aortas of the rats were excised and immersed in ice-cold, modified Krebs solution. The aortas were cleaned of all connective tissue, soaked in Krebs-bicarbonate solution, and cut into four ring segments (3.5 mm long). Each aortic ring was suspended in a water-jacketed organ bath (6 mL) maintained at 37°C and aerated with a mixture of 95% O_2_ and 5% CO_2_. Each ring was connected to an isometric force transducer (Danish Myo Technology, Skejbyparken, Aarhus N, Denmark). The rings were stretched to an optimal resting tension of 1.0 or 2.0 g, which was maintained throughout the experiment. Each ring was equilibrated in the organ bath solution for 90 min before the contractile responses were measured after the addition of 50 mM KCl. To determine the effect of MS-275 on the maintenance of vascular tension in rat endothelium-intact or -denuded aortic rings, vascular contractions were induced using the thromboxane A2 agonist, U46619 (30 nM, 20 min). When each contraction plateaued, MS-275 was added cumulatively (0.003–3 μM) to elicit vascular relaxation.

In the second experiment, we investigated the inhibition of the relaxation response by treating endothelium-intact aortic rings with N^G^-nitro-L-arginine methyl ester (_L_-NAME, 10 and 100 μM) for 30 min. After U46619 treatment, MS-275 was cumulatively (0.003–3 μM) added to the aortic rings.

### Measurement of NO production in vascular smooth muscle cells (VSMCs), the aorta and in serum

To measure the amount of NO in VSMCs, we performed a Griess assay as described previously [30]. VSMCs were seeded in 12 well plates. Cells were starved from serum for 1 day and incubated with MS-275 (3 and 10 μM), with or without angiotensin II (1 μM) for 24 h. Hundred microliters of supernatant of cells were added into a 96-well plate, and 50 μl of 1% sulfanilamide in 5% phosphoric acid was added in the dark. Fifty microliters of 0.1% N-1-naphthylethylenediamine dihydrochloride in water were added to the plate for 10 min, and absorbance was measured at 570 nm. NO levels in the serum (100 μl) were measured without dilution. Aorta NO levels were determined using 50 μg of protein, which was deproteinized with 5% trichloroacetic acid (TCA).

### Quantitative real-time polymerase chain reaction (qPCR)

Total RNA from the aorta tissue and cells was isolated with TriZol reagent (Invitrogen Life Technologies, Waltham, MA, USA), and 1 μg RNA was used for the reverse transcription (RT) reaction using TOPscript RT DryMIX (Enzynomics, Daejeon, South Korea). The mRNA amounts were determined using the SYBR Green PCR kit (Enzynomics, Daejeon, South Korea). All the data were normalized to the glyceraldehyde 3-phosphate dehydrogenase (GAPDH) expression using the 2-^ΔΔct^ method. The PCR primers used in this study are shown in Table 1 and Table 2.

**Table 1:** Primers for reverse transcription-polymerase chain reaction (RT-PCR).

Gene	Primer sequence (5ʹ to 3ʹ)
*GAPDH* (mouse)	F: GCATGGCCTTCCGTGTTCCT R: CCCTGTTGCTGTAGCCGTATTCAT
*AT1* (mouse, rat)	F: GGAAACAGCTTGGTGGTGAT R: GGCCGAAGCGATCTTACATA
*ACE1* (mouse)	F: CAGTGTCTACCCCCAAGCAT R: TTCCATCAAAGACCCTCCAG
*eNOS* (mouse)	F: AAGTGGGCAGCATCACCTAC R: GAGCCACTCCTTTTGATGGA
*iNOS* (mouse)	F: CTCACTGGGACAGCACAGAA R: TGGTCAAACTCTTGGGGTTC
*GTPCH (mouse)*	F: TCACCAAGGGATACCAGGAG R: AGCCAATATGGACCCTTCCT
*Cox-2 (mouse)*	F: ATCCTGAGTGGGGTGATGAG R: GGCAATGCGGTTCTGATACT
*Arginase 1 (mouse)*	F: TCACCTGAGCTTTGATGTCG R: CACCTCCTCTGCTGTCTTCC
*Arginase 2 (mouse)*	F: ACCAGGAACTGGCTGAAGTG R: CATGAGCATCAACCCAGATG
*Nox1 (mouse*, *rat)*	F: ACCAGGAACTGGCTGAAGTG R: CATGAGCATCAACCCAGATG
*Nox2 (mouse*, *rat)*	F: TGTCATTCTGGTGTGGTTGG R: GAACCCCTGAGGAAGGAGAG
*Nox4 (mouse*, *rat)*	F: CTGGAAGAACCCAAGTTCCA R: ACTGGCCAGGTCTTGCTTTA
*P22phox (mouse*, *rat)*	F: AAAGAGGAAAAAGGGCTCCA R: CTGCCAGCAGGTAGATCACA

**Table 2:** Primers for reverse transcription-polymerase chain reaction (RT-PCR).

Gene	Primer sequence (5ʹ to 3ʹ)
*SOD3* (mouse)	F: TTGGCCTTCTTGTTCTACGG R: ATGCGTGTCGCCTATCTTCT
*NF-kB p65* (mouse)	F: TCAATGGCTACACAGGACCA R: CTAATGGCTTGCTCCAGGTC
*TNF-α* (mouse)	F: AGCCCCCAGTCTGTATCCTT R: CTCCCTTTGCAGAACTCAGG
*TGF-β* (mouse)	F: TTGCTTCAGCTCCACAGAGA R: TGGTTGTAGAGGGCAAGGAC
*IL-1β* (mouse)	F: GCCCATCCTCTGTGACTCAT R: AGGCCACAGGTATTTTGTCG
*VCAM-1 (mouse)*	F: TCATCCCCACCATTGAAGAT R: TGAGCAGGTCAGGTTCACAG
*ICAM-1 (mouse)*	F: CCTGTTTCCTGCCTCTGAAG R: TTAAGGTCCTCTGCGTCTCC
*Cyclin D1 (mouse)*	F: AACTACCTGGACCGCTTCCT R: CCACTTGAGCTTGTTCACCA
*Cyclin E1 (mouse)*	F: GTCTGAGTTCCAAGCCCAAG R: CAGGGCTGACTGCTATCCTC
*E2F3 (mouse)*	F: ATCCAAAGCTGTACCCTGGA R: TGGGTACTTGCCAAATGGAT
*GATA6 (mouse)*	F: GAGGACCTGTCGGAGAGCCG R: GCAAGTGGTCGAGGCACCCC
*ANP (mouse)*	F: TGGAGGAGAAGATGCCGGTAGAAGAT R: AGCGAGCAGAGCCCTCAGTTTGCT
*BNP (mouse)*	F: CTGAAGGTGCTGTCCCAGAT R: GTTCTTTTGTGAGGCCTTGG
*Skeletal α-actin (mouse)*	F: CGACATCAGGAAGGACCTGT R: ACATCTGCTGGAAGGTGGAC
*ACE1 (rat)*	F: GTACAGAAGGGCTGGAATGC R: CGTGCACTGCTTAATCCTGA
*eNOS (rat)*	F: TGGCAGCCCTAAGACCTATG R: AGTCCGAAAATGTCCTCGTG
*SOD3 (rat)*	F: GAGTCCGGTGTCGACTTAGC R: GGACCAAGCCTGTGATCTGT

F, forward; R, reverse.

### Drugs

Ang II was obtained from EMD Millipore (Billerica, MA, USA). MS-275 (S1053) and RGFP966 (S7229) were purchased from Selleckchem (Houston, TX, USA).

### Statistics

The statistical analysis was performed using a one-way analysis of variance (ANOVA) followed by the Bonferroni post-hoc test for comparative analysis between the treatment groups (GraphPad Prism, version 5.0; GraphPad Software, La Jolla, CA, USA). The data are presented as the means ± standard error (SE). A P < 0.05 was considered statistically significant.

## Results

### MS-275 reduces blood pressure in Ang II-induced hypertensive mice by downregulating AT1 and angiotensin-converting enzyme 1 (ACE1)

To investigate whether class I HDAC is implicated in the regulation of hypertension, we evaluated the antihypertensive effect of both class I HDAC inhibitors in Ang II-induced hypertensive mice. MS-275 (entinostat) is a benzamide derivative of an HDAC inhibitor.

**Fig 1:**
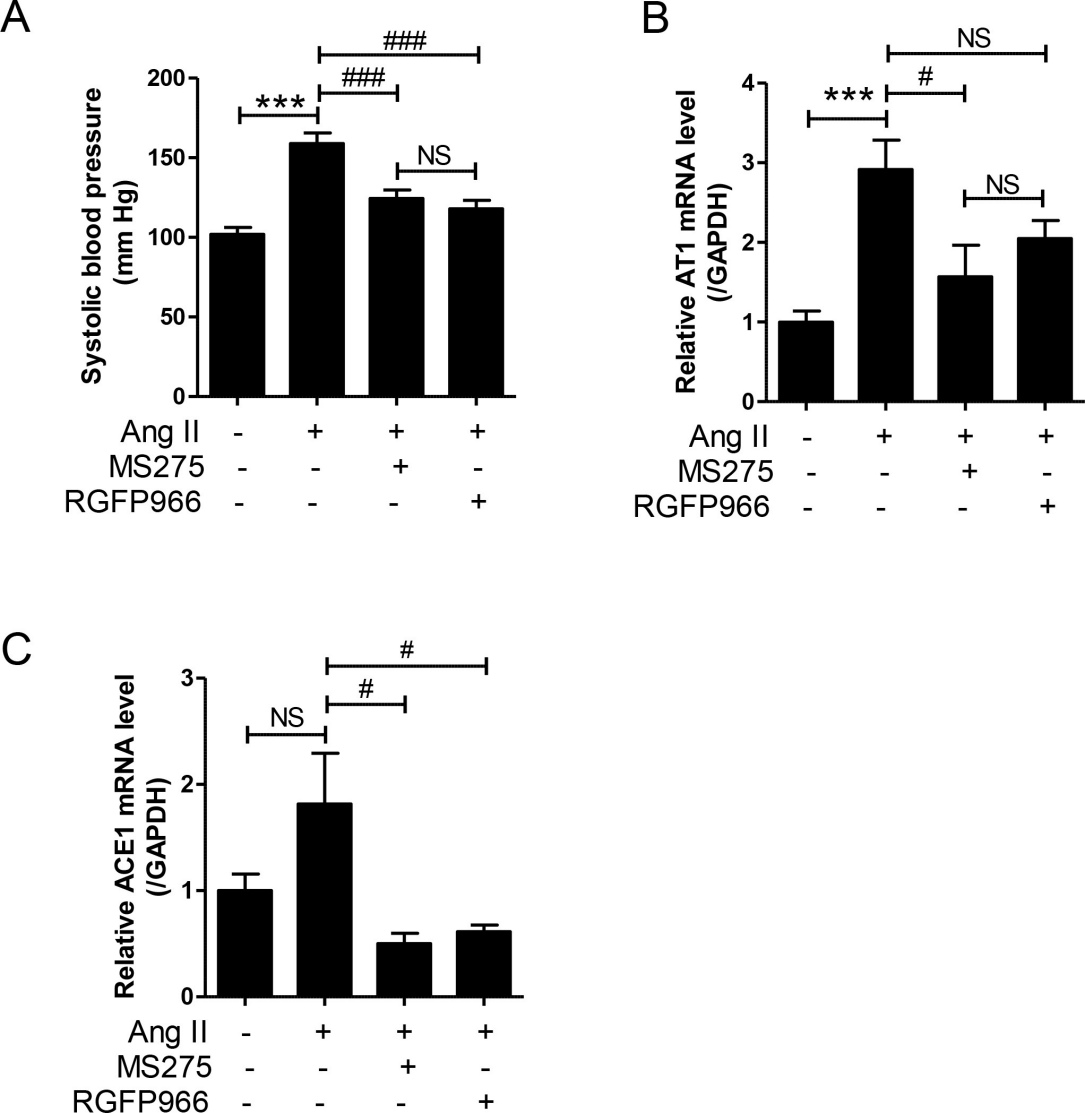
MS-275 and RGFP966 reduced systolic blood pressure in angiotensin II-induced hypertensive mice by downregulating AT1 and angiotensin-converting enzyme 1 (ACE1) expression. (A) After angiotensin II infusion (1.3 mg·kg^-1^·day^-1^) to mice for 1 week, we injected MS-275 or RGFP966 (both 3 mg·kg^-1^·day^-1^) daily to mice for additional 7 days. Systolic blood pressures were measured in awake mice. (B and C) Transcript levels of aortic AT1 and ACE1 were quantified using quantitative real-time reverse transcription-polymerase chain reaction (qRT-PCR). GAPDH was used to normalize the values. Data are presented as the means ± SE (n = 8 per group). ***p < 0.001 versus sham group; ^#^p < 0.05 and ^###^p < 0.001 versus angiotensin II group; NS, not significant.

Measurement of HDAC enzyme activity in the cell-free system revealed that MS-275 effectively inhibited HDAC1, 2, and 3 (IC_50_ 0.228, 0.364, and 0.744 μM, respectively, Table 3). RGFP966 is known to inhibit HDAC3 selectively [31]. Indeed, we observed that it weakly inhibited HDAC3 (IC_50_ 2.686 μM, Table 3). As shown in Fig 1A, Ang II infusion increased the systolic blood pressure to approximately 160 mmHg. Both class I HDAC inhibitors MS-275 and RGFP966 significantly reduced systolic blood pressure in Ang II-infused mice. No difference was observed in the systolic blood pressure between MS-275 and RGFP966 treatments. Ang II activates the AT receptor and induces aldosterone, which in turn, promotes angiotensin-converting enzyme (ACE1) [32]. To determine whether HDAC inhibitors affect the RAS, we performed qRT-PCR. Ang II infusion significantly increased the mRNA levels of AT1 in the aortas compared to that in the sham group. This increase was decreased by MS-275 but not RGFP966 (Fig 1B).

**Table 3:** IC_50_ [μM] values for TMP269, RGFP966, and TSA.

compound	HDAC1	HDAC2	HDAC3	HDAC8
MS-275	0.228	0.364	0.744	>10
RGFP966	> 10	>10	2.686	>10
TSA	0.002	0.006	0.003	0.669

The in vitro inhibitory activity of MS-275 and RGFP966 against each HDAC isoform was determined by using the HDAC Fluorogenic Assay Kit from BPS Bioscience. The IC_50_ values were determined using 0.0001, 0.0003, 0.01, 0.03, 0.1, 0.3, 1, 3, and 10 μM of an inhibitor. TSA was used as the reference compound.

In contrast, ACE1 mRNA levels were not increased in response to Ang II but were significantly reduced by MS-275 treatment (Fig 1C). There were no significant differences in AT1 and ACE1 mRNA levels between MS-275 and RGFP966 treatment groups.

### MS-275 and RGFP966 reduces cardiac hypertrophy in Ang II-induced mice

**Fig 2:**
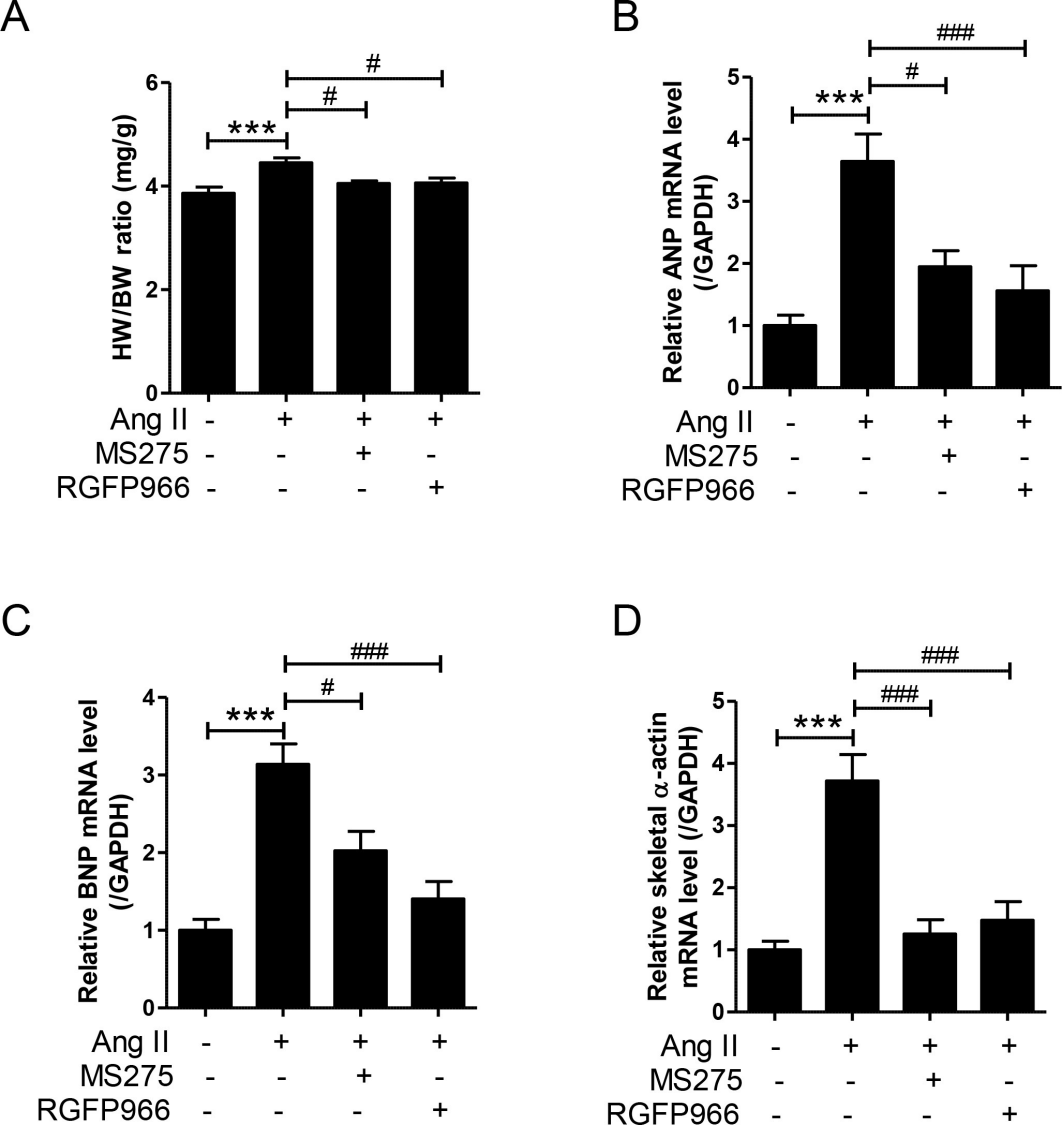
MS-275 and RGFP966 reduced cardiac hypertrophy in angiotensin II-induced hypertensive mice. (A) The HW/BW ratio was analyzed 2 weeks after Ang II infusion (n = 8 per group). ***p < 0.001 versus sham group; ^#^p < 0.05 versus angiotensin II (Ang II) group. (B‒D) ANP, BNP, and skeletal α-actin mRNA was estimated by qRT-PCR. ***p < 0.001 versus sham group; ^#^p < 0.05 and ^###^p < 0.001 versus angiotensin II group.

To determine whether HDAC inhibitors suppress cardiac hypertrophy, heart weight to body weight (HW/BW) ratio and cardiac hypertrophic markers were estimated. As shown in Fig 2A, MS-275 and RGFP966 administration reduced the HW/BW ratio induced by Ang II infusion in mice. MS-275 and RGFP966 treatment significantly reduced the mRNA levels of cardiac hypertrophic markers, such as atrial natriuretic peptide (ANP) and brain natriuretic peptide (BNP), and skeletal a-actin in heart tissues of Ang II mice (Fig 2B–2D).

### MS-275 and RGFP966 reduces aortic wall thickness in Ang II-induced hypertensive mice by suppressing E2F transcription factor 3 (E2F3) and GATA binding protein 6 (GATA6) expression

**Fig 3:**
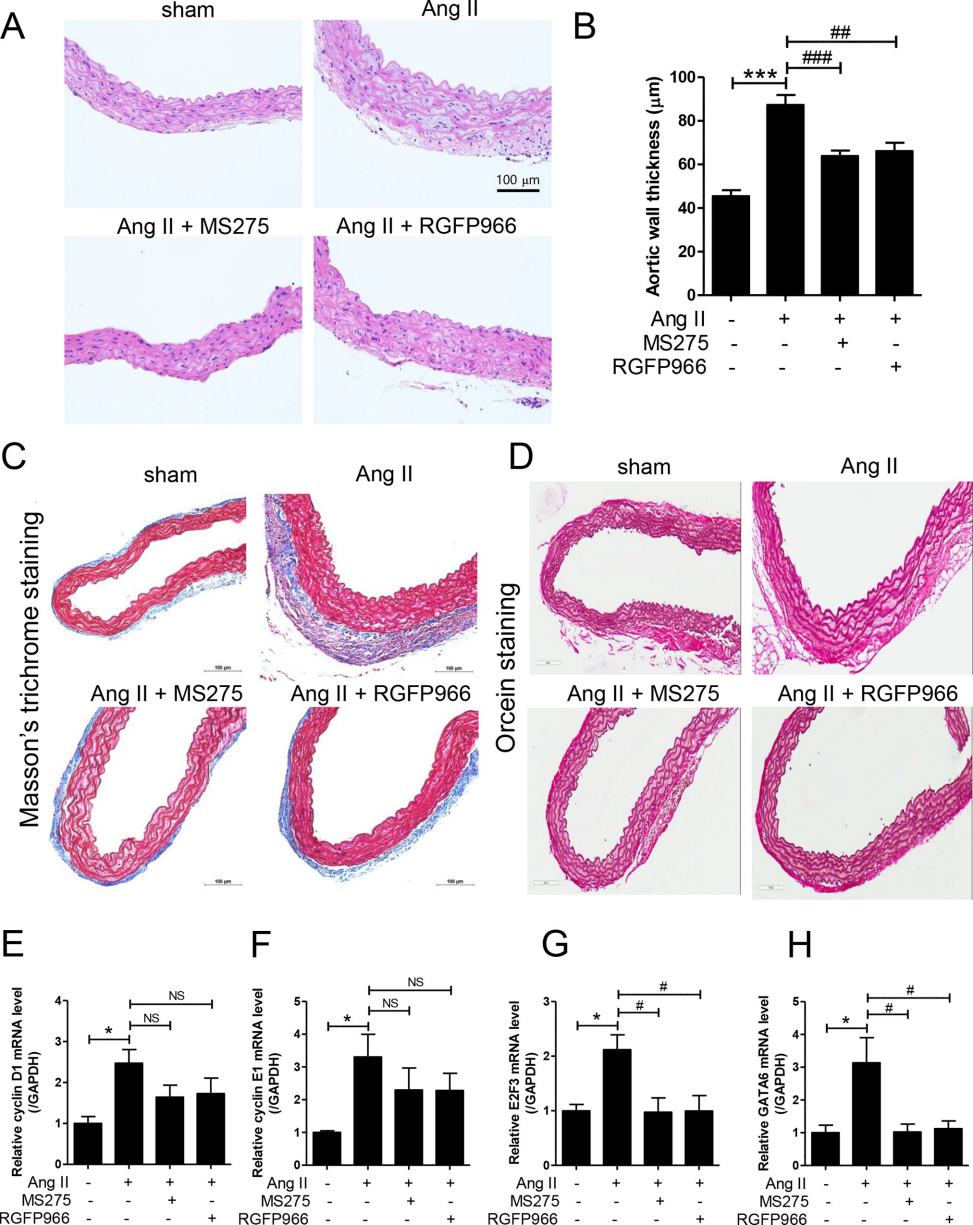
MS-275 and RGFP966 reduced arterial wall thickness in angiotensin II-induced hypertensive mice by suppressing E2F3 and GATA6 expression. (A) Representative images of H&E stained aortas from sham, angiotensin II group (Ang II), MS-275-treated angiotensin II group (Ang II + MS-275), and RGFP966-treated angiotensin II group (Ang II + RGFP966). Scale bar = 100 μm. (B) Arterial wall thickness was quantified. Data are means ± SE (n = 7/group). ***p < 0.001 versus sham group; ^##^ p < 0.01 and ^###^ p < 0.001 versus angiotensin II group. (C‒D) Masson’s trichrome and orcein staining of representative aorta sections. Scale bar = 100 μm. Collagen deposition in aorta is shown as blue staining. (E‒H) The transcript levels of cyclin D1, cyclin E1, E2F3, and GATA6 were quantified using quantitative real-time reverse transcription-polymerase chain reaction (qRT-PCR). * p < 0.05 versus sham group; ^#^ p < 0.05 versus angiotensin II group; NS, not significant.

Ang II induces the development of vascular remodeling and hypertension [33]. To identify whether class I HDAC inhibitors regulate vascular remodeling, we measured aortic wall thickness after H&E staining of aortic tissue. As shown in Fig 3A, aortic wall thickness was increased by approximately two times in Ang II mice (87 μm) over the sham group (45 μm). MS-275 and RGFP966 HDAC inhibitors significantly decreased the enlarged aortic wall thickness in Ang II-induced mice (Fig 3B). To further determine whether HDAC inhibitors affect the extracellular matrix, we performed Masson’s trichrome staining and orcein staining on the aortic tissues. MS-275 treatment attenuated collagen deposition in the adventitia of aortic tissues (Fig 3C). MS-275 reduced the collagen type III mRNA levels in Ang II mice (S1 Fig). Orcein staining demonstrated that there was no difference in elastic fiber structure between the four groups (Fig 3D).

To determine whether cell cycle-related genes are involved in the vascular hypertrophy, we performed qRT-PCR using aortic tissues. The results showed that mRNA levels of cyclin D1 and cyclin E1 increased in the aortic tissues of Ang II-treated mice but were not significantly decreased by MS-275 and RGFP966 treatment (Fig 3E and 3F). The E2F transcription factor 3 (E2F3) mRNA levels also increased in response to Ang II, and this was significantly inhibited by MS-275 and RGFP966 treatment (Fig 3G). Forced expression of GATA binding protein 6 (GATA6) directly caused vascular remodeling [26]. Ang II infusion increased GATA6 mRNA level in aortic tissue. The increase was significantly suppressed by MS-275 and RGFP966 administration (Fig 3H).

### MS-275 induces vascular relaxation via a NO-dependent pathway ex vivo and in VSMCs

**Fig 4:**
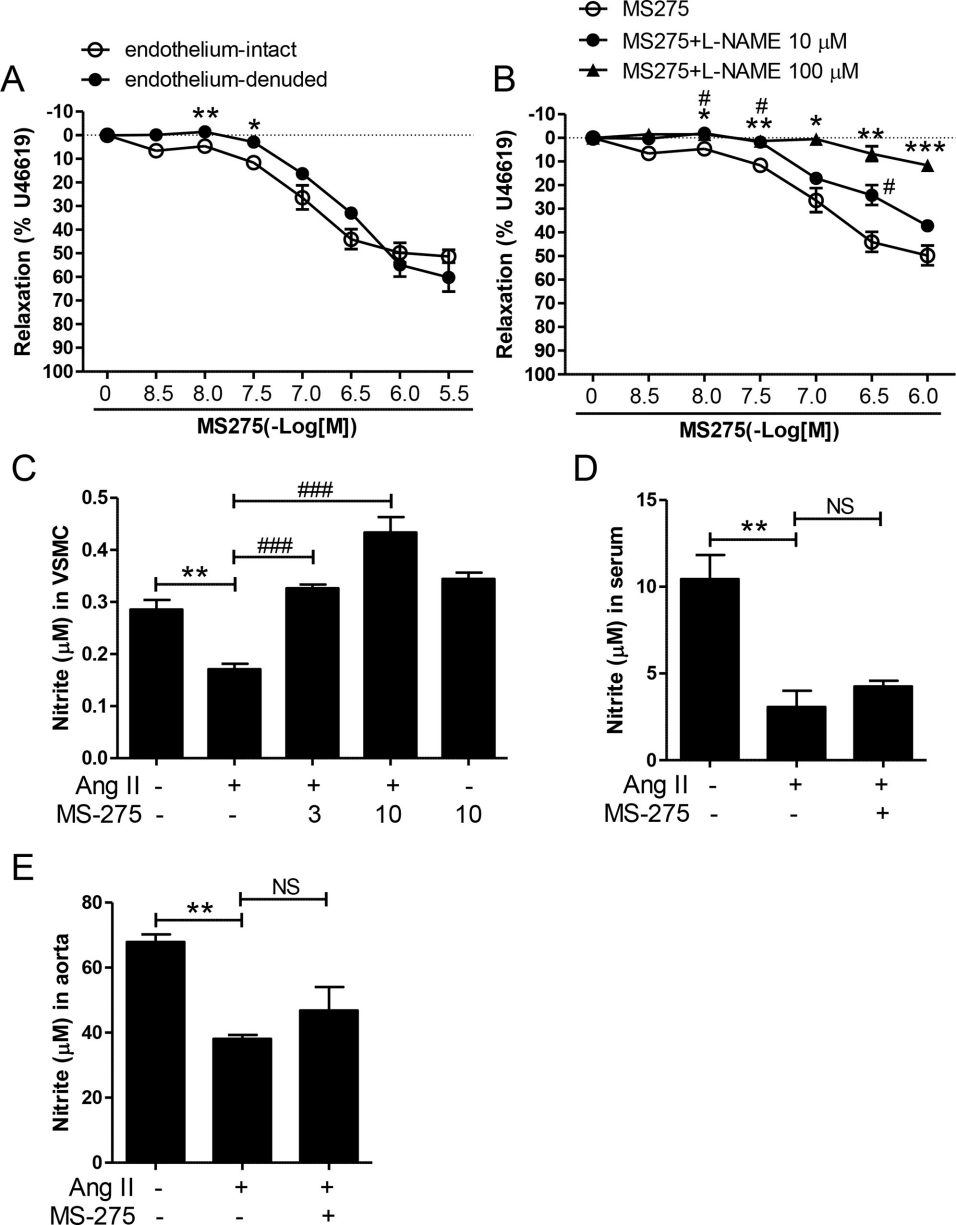
MS-275 induced vascular relaxation via NO production. (A) U46619-precontracted endothelium-intact and -denuded aortic rings were relaxed by cumulative concentrations of MS-275. (B) Concentration-response curves of endothelium-intact aortic rings were precontracted with U46619 and incubated with MS-275 in the absence or presence of the NO synthase inhibitor _L_-NAME (10 and 100 μM). (C) VSMCs were treated with MS-275 (10 and 100 μM) or vehicle in the presence or absence of angiotensin II (1 μM) for 24 h. NO production in VSMC medium was evaluated using Griess reagent. The values are the means ± SE of three independent experiments. ** p < 0.01 versus vehicle-treated group; ^###^ p < 0.001 versus angiotensin II group. NO production in serum (D) or aortic tissues (E) from sham, angiotensin II group (Ang II), and MS-275-treated angiotensin II group (Ang II + MS-275).

To determine whether the blood pressure-lowering effect of MS-275 is regulated by vascular relaxation, we investigated the vasoconstriction-relaxation response in rat aortic rings. We found that 30 nM U46619 caused sustained contractions in aortic rings. MS-275 treatment induced vasorelaxation of both endothelium-intact and -denuded aortas (Fig 4A). The maximum relaxation was observed following 3 μM MS-275 treatment. The vascular relaxation induced by MS-275 treatment seemed to be unaffected whether the endothelium was intact or not (Fig 4A). To further explore whether MS-275 induces vascular relaxation by regulating the NO pathway, we pretreated endothelium-intact aortic rings with _L_-NAME, 10 or 100 μM) or the vehicle for 30 min. As shown in Fig 4B, _L_-NAME pretreatment reduced MS-275-induced vascular relaxation in endothelium-intact aortic rings. The higher concentration (100 μM) of _L_-NAME showed greater inhibition than the low concentration (10 μM) did. To determine whether MS-275 causes aortic vessel relaxation, we measured NO production using Griess reagent in VSMCs. Ang II treatment reduced NO levels in a VSMC medium compared to a vehicle-treated control group (Fig 4C). Treating VSMCs with 3 and 10 μM concentrations of MS-275 significantly restored a lower NO level. NO levels were also significantly reduced in serum and in the aorta of Ang II infused mice (Fig 4D and 4E). However, NO levels were not induced by MS-275 treatment.

### MS-275 and RGFP966 does not regulate eNOS uncoupling in vivo

**Fig 5:**
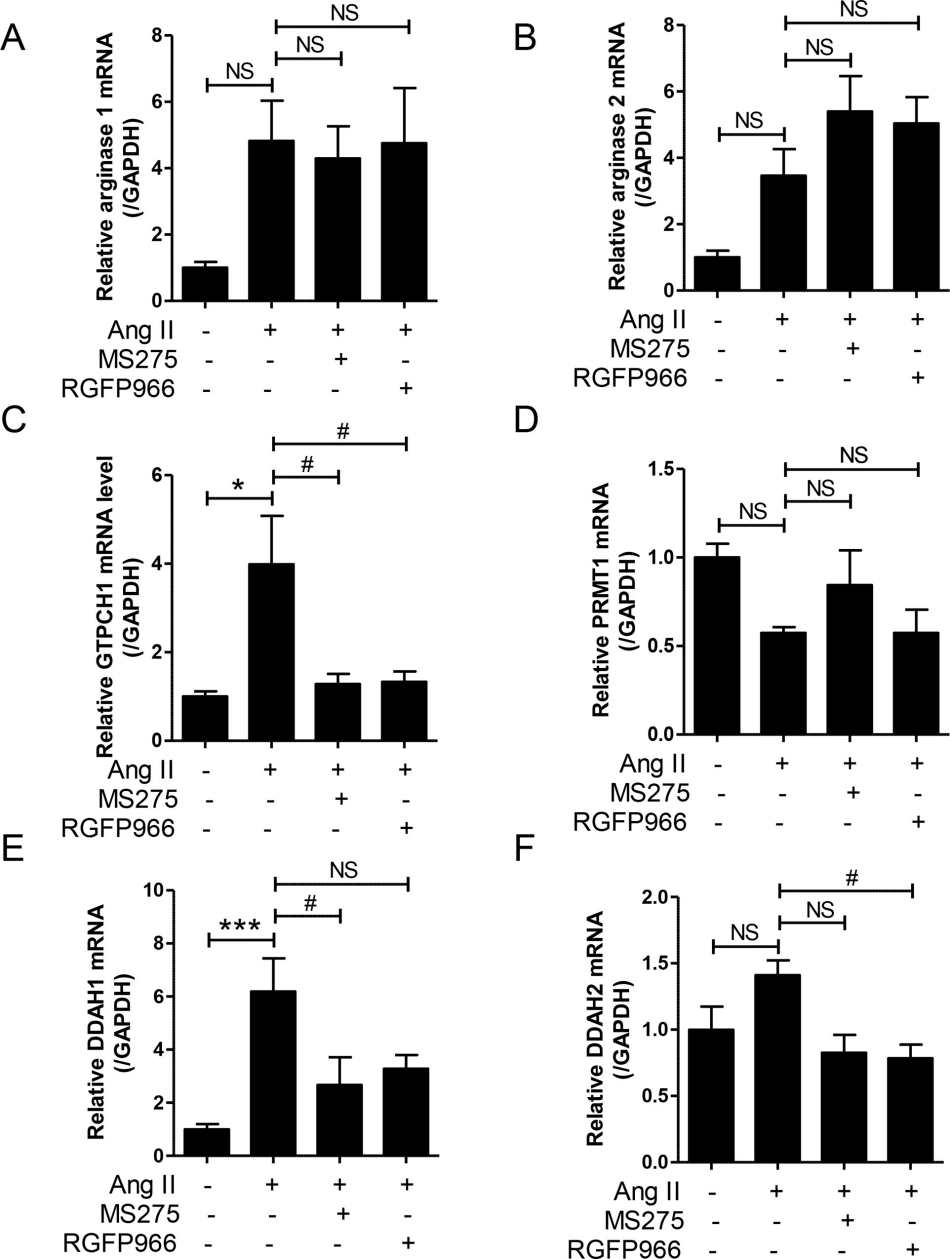
MS-275 and RGFP966 did not regulate eNOS uncoupling. The transcript levels for arginase 1 (A), arginase 2 (B), GTPCH1 (C), PRMT1 (D), DDAH1 (E), and DDAH2 (F) were determined using quantitative real-time reverse transcription-polymerase chain reaction (qRT-PCR) in aortas of sham and angiotensin II treated with vehicle, MS-275, or RGFP966. Data are presented as the means ± SE (n = 8 per group). *p < 0.05 and ***p < 0.001 versus sham group; ^#^p < 0.05 versus angiotensin II group; NS, not significant.

Hypertension is characterized by endothelial dysfunction with eNOS uncoupling [34]. Therefore, we examined the gene expression of the substrates that regulate eNOS. Arginase upregulation is associated with eNOS uncoupling and contributes to endothelial dysfunction [35]. The mRNA levels of arginase 1 and 2 were approximately increased by four-fold in Ang II-treated aortic tissue compared to the sham group (Fig 5A and 5B). However, this increase was not reduced by treatment with MS-275 and RGFP966. Tetrahydrobiopterin (BH_4_) is an essential cofactor for eNOS in the production of NO [16]. GTP cyclohydrolase 1 (GTPCH1) is involved in BH_4_ biosynthesis pathway [36]. As shown in Fig 5C, GTPCH mRNA levels were significantly increased in Ang II-treated aortic tissue compared with the sham group. The expression was significantly reduced by MS-275 and RGFP966 administration (Fig 5C). Asymmetric dimethylarginine (ADMA) is an endogenous eNOS inhibitor, which is increased in hypertension [37, 38]. It is generated by protein arginine N-methyltransferase 1 (PRMT1) enzyme. However, unexpectedly, PRMT1 mRNA levels were decreased in Ang II-induced mice compared to the sham group. HDAC inhibitors did not affect the mRNA levels of PRMT1 (Fig 5D). Dimethylarginine dimethylaminohydrolase 1 (DDAH1) is the critical enzyme for degrading ADMA [39]. DDAH1 mRNA levels were significantly increased by Ang II treatment and suppressed by MS-275 treatment (Fig 5E). In contrast, the expression of DDAH2 mRNA was not significantly increased in response to Ang II stimulus (Fig 5F).

### MS-275 and RGFP966 does not regulate oxidative stress in Ang II-induced hypertensive mice

**Fig 6:**
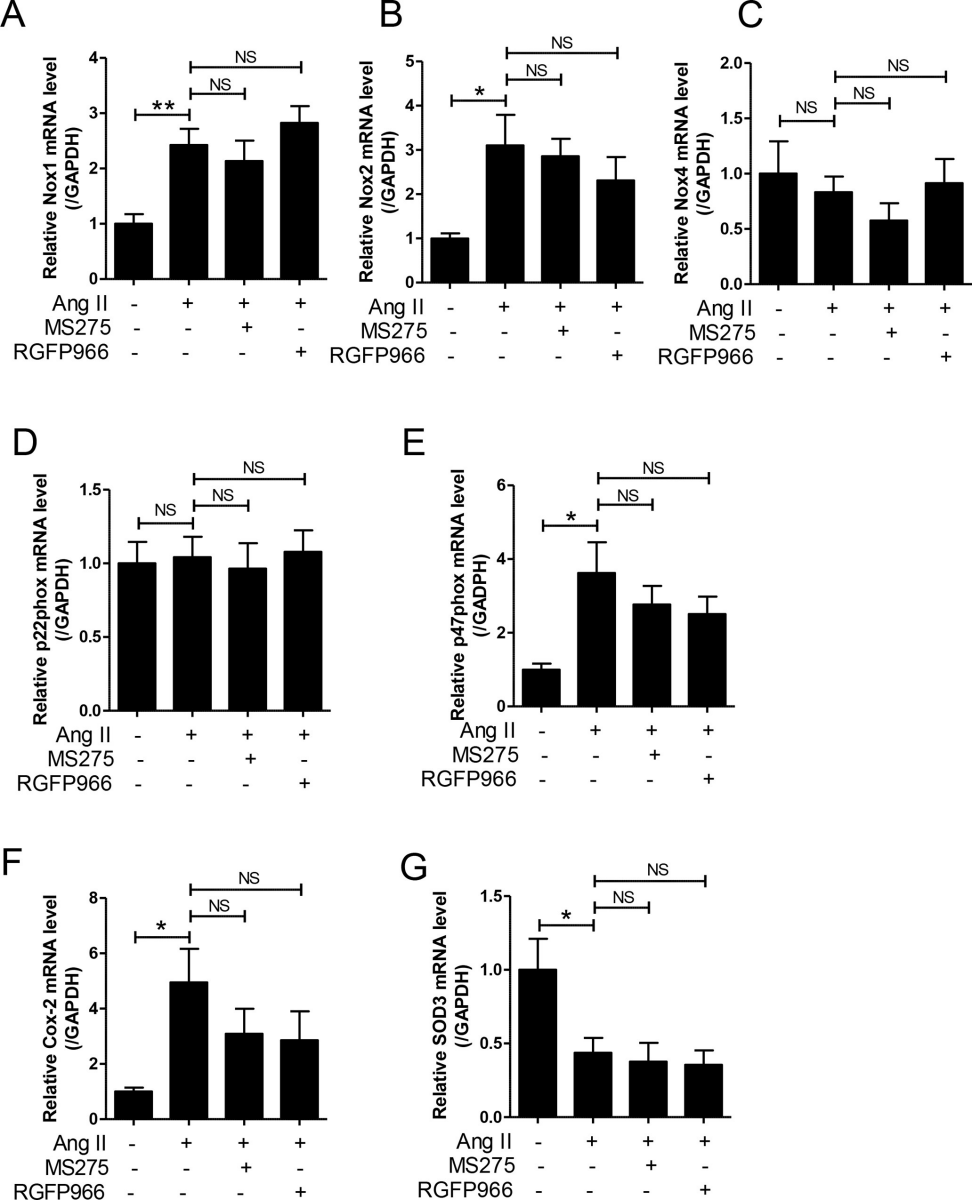
MS-275 and RGFP966 did not reduce oxidases and restore antioxidant enzymes in angiotensin (Ang) II-induced hypertensive mice. The transcript levels of Nox1 (A), Nox2 (B), Nox4 (C), p22phox (D), p47phox (E), Cox-2 (F), and SOD3 (G) were determined using qRT-PCR in aortas of sham and Ang II-induced mice treated with vehicle, MS-275, or RGFP966. Results are ± SE (n = 8 per group). * p < 0.05 and ** p < 0.01 versus sham group; NS, not significant.

Oxidative stress such as increased reactive oxygen species (ROS) is implicated in hypertension. We examined the effect of HDAC inhibitors on the expression of Nox isoforms and their regulatory subunits. Ang II treatment significantly increased the mRNA levels of Nox1, Nox2, and p47phox, whereas it did not induce the mRNA levels of Nox4 and p22phox (Fig 6A–6E). The expression levels of Nox1, Nox2, and p47phox mRNA were unchanged by MS-275 and RGFP966 treatment. Cox-2 is a source of ROS and is implicated in inflammation. We observed that Cox-2 mRNA levels were increased in the aortas of Ang II-treated mice compared to that in the sham group. However, this increase was not significantly decreased by MS-275 and RGFP966 administration (Fig 6F). To further determine whether HDAC inhibitors affect antioxidant enzymes, we examined the expression of SOD3. In contrast to the upregulation of Nox, the mRNA levels of SOD3 were significantly reduced in response to Ang II in vivo (Fig 6G). However, MS-275 and RGFP966 treatment did not restore the expression of SOD3 in the aortas from Ang II-treated mice.

### MS-275 reduces inflammation in Ang II-induced hypertensive mice

**Fig 7:**
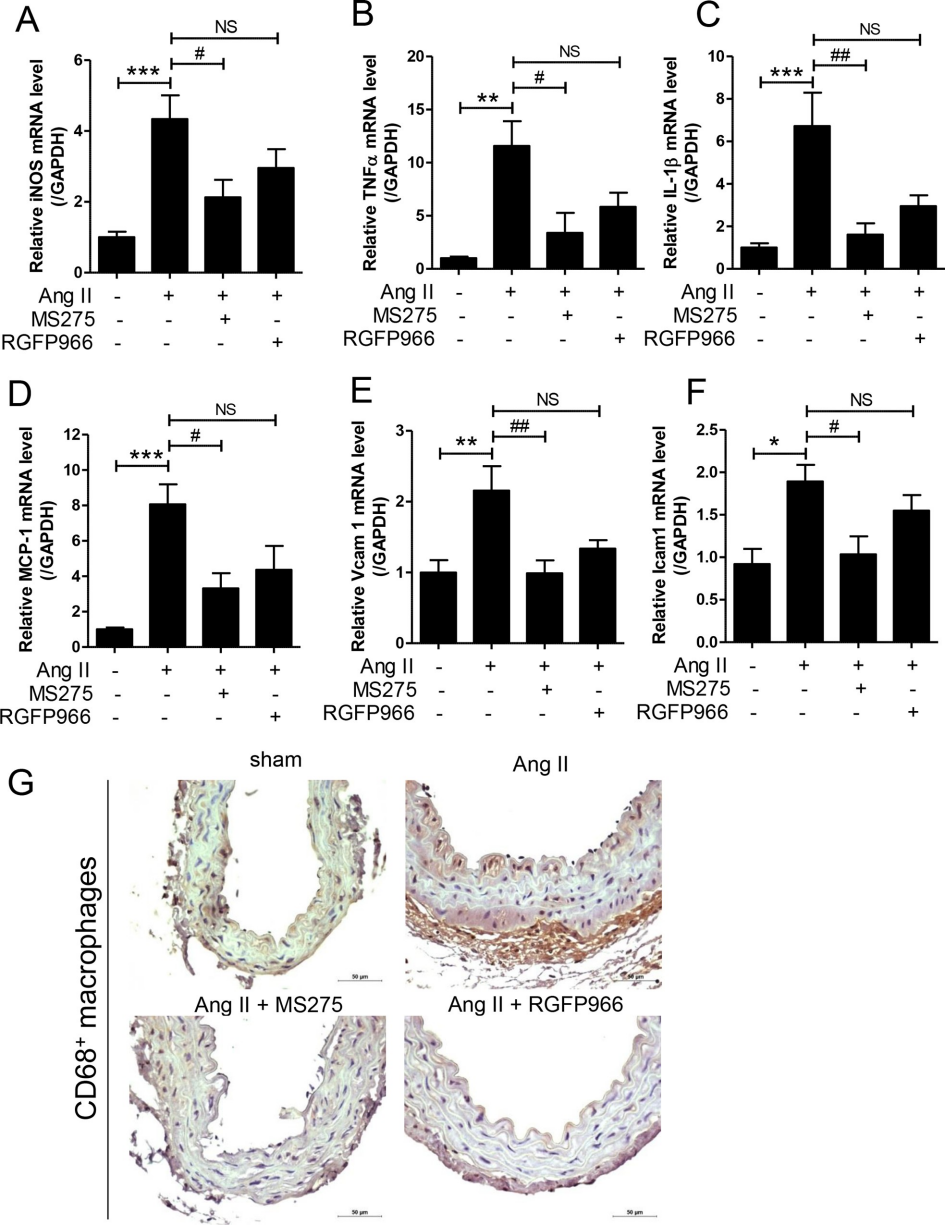
MS-275 reduced inflammation in angiotensin (Ang) II-induced hypertensive mice. The transcript levels of iNOS (A), TNF-α (B), IL-1β (C), MCP-1 (D), VCAM-1 (E), and ICAM-1 (F) were determined using quantitative real-time reverse transcription-polymerase chain reaction (qRT-PCR) in aortas of sham and Ang II-induced mice treated with vehicle, MS-275, or RGFP966. Results are means ± SE (n = 8 per group). * p < 0.05, ** p < 0.01, and *** p < 0.001 versus sham group; ^#^ p < 0.05 and ^##^ p < 0.01 versus angiotensin II group; NS, not significant. (G) Representative aortic images for macrophage infiltration are shown. Scale bar = 50 μm.

To investigate the anti-inflammatory effect of MS-275 and RGFP966 in hypertension, we performed qRT-PCR using aortic tissues. Ang II infusion increased the mRNA levels of iNOS, TNF-α, IL-1β, and MCP-1 compared to the sham group. The expression was significantly attenuated by MS-275 but not RGFP966 administration (Fig 7A–7D). Adhesion molecules are implicated in inflammation. Therefore, we next examined the adhesion molecules. MS-275 but not RGFP966 treatment significantly inhibited Ang II-induced expression of VCAM-1 and ICAM-1 in mice (Fig 7E and 7F). We investigated inflammatory cell infiltration to further show the anti-inflammatory effect of HDAC inhibitors. As shown in Fig 7G, the number of CD68^+^ macrophages increased in the aorta tissue of Ang II mice compared to that in the sham group. In particular, the recruitment of CD68^+^ cells was observed in the adventitia and the media. In the negative control, no staining was seen in the aortic tissues, including the adventitia (S2 Fig). This increase was reduced in Ang II mice treated with MS-275 and RGFP966.

## Discussion

In our current study, we found that MS-275, a class I HDAC-selective inhibitor (HDAC1, HDAC2, and HDAC3), lowered blood pressure, reduced blood vessel thickness, and inhibited inflammation in an Ang II-induced hypertensive animal model. The antihypertensive effects of RGFP966, an HDAC3-selective inhibitor, were compared with those of MS-275. We found that the two drugs, MS-275 and RGFP966, similarly lowered blood pressure. RAAS is a central pathway involved in the regulation of blood pressure. In particular, MS-275 reduced the aortic expression of ACE1 and AT1, suggesting that class I HDAC inhibition affects the RAAS, decreasing the blood pressure. Very few studies on hypertensive treatments using HDAC inhibitors have been conducted. Long-term treatment with a pan-HDAC inhibitor, such as valproate, attenuated hypertension in SHR by downregulating cardiac AT1 mRNA and protein expression [40]. In addition, valproic acid prevents high-fat diet-induced hypertension by inhibiting AT1 expression. Similar to our findings, the antihypertensive effects of valproic acid and valproate seem to be due to the inhibition of AT1 expression. However, the expression of AT1 was not investigated in the study of MS-275, which inhibited blood pressure in Cushing’s syndrome [23]. AT1 and ACE expression was reported to be higher in the aorta of SHR rats than in that of WKY control rats [30]. However, under our experimental conditions, the expression of ACE1 did not increase significantly after Ang II administration to mice. This finding contradicts that of Koka et al. [32], who showed that ACE1 expression was upregulated in human hypertensive kidney and Ang II-treated human kidney tubular epithelial cell line (HK-2). The discrepancy in the results can be attributed to the use of mouse versus human and aortic vessel versus kidney.

Hypertension is related to the structural changes in blood vessels called remodeling [41]. Ang II is a vasoconstrictor hormone, which induces vascular remodeling in vivo [33, 42]. Ang II affects vascular remodeling in the heart and kidneys [26, 43]. Ang II-induced aortic vessel remodeling was improved by the HDAC inhibitors MS-275 and RGFP966. This regulatory action is likely attributed to the downregulation of E2F3 and GATA6 expression. Similar to this result, we previously reported that MC1568, an HDAC inhibitor, reduced the increase in vascular thickness induced by Ang II and reduced GATA6 expression [26]. Similarly, one study group reported that HDAC inhibition suppressed hypoxia-induced VSMC proliferation [44]. HDAC inhibitors are known to inhibit the proliferation of various cancer cells [45]. Therefore, taken together, our findings indicate that HDAC inhibitors are effective in inhibiting abnormal proliferation that leads to the growth of cancer cells and VSMC after vascular injury.

In vascular reactivity experiments, MS-275 increased the relaxation of vascular contraction in rat aortic rings. However, there was little difference in vascular relaxation between the presence and absence of vascular endothelial cells. These findings suggest that VSMCs are more important to the relaxation reaction by MS-275 than endothelial cells. It also indicates that the class I HDAC-selective inhibitor improved vascular function. Similarly, TSA also reduced hypertension induced by abdominal aortic coarctation, while TSA inhibited Ang II-induced vascular contraction in the rat aorta [25]. In the present study, the regulatory action of the vascular relaxation by MS-275 is likely to have been mediated by NO signaling. This is likely because the vascular relaxation caused by MS-275 was blocked by _L_-NAME pretreatment. VSMCs played an important role in vascular relaxation by MS-275; MS-275 dose-dependently increased NO production in vitro. The generation of NO in VSMCs is mainly caused by iNOS. Additionally, NO production was decreased in the aorta and serum from Ang II-infused mice; however, it was not increased by MS-275. The MS-275 doses used in our animal experiments may not have been enough to increase NO production. The inconsistency between the aorta’s NO production and the expression of iNOS can be explained by inflammatory cytokines; iNOS is increased by cytokines such as IL-1β, as well as Ang II [46].

We investigated eNOS uncoupling. Indeed, arginase 1 and 2 mRNA expression exhibited an increasing tendency in hypertension, which was not affected by class I HDAC inhibitors. The increase in aortic arginase 1 and 2 indicated a reduction in available L-arginine required to synthesize NO, indicating a decrease in eNOS-induced NO formation. In fact, vascular arginase is upregulated in hypertension [47]. Arginase is known to promote endothelial dysfunction [48]. The pharmacological blockade of arginase reduces blood pressure increases in SHR [49], implying that arginase plays an important role in NO production. Furthermore, long-term treatment with an arginase inhibitor improved vascular function and reduced cardiac fibrosis in SHR [50]. In addition, eNOS uncoupling-related enzymes showed unexpected changes in expression in Ang II-induced hypertension.

In this study, class I HDAC inhibitors did not show positive effects on oxidative stress in hypertension. Nox1, Nox2, and p47phox mRNA expression levels were significantly increased in aortic tissues of the Ang II treatment group. However, MS-275 and RGFP966 did not suppress this effect. Conversely, the expression of *SOD3*, an antioxidant stress gene, was reduced during Ang II treatment but was not recovered by class I HDAC inhibitors. However, HDAC inhibitors including TSA, SAHA, Scriptaid, and valproate reduced Nox1 and Nox2 expression in human immune cell lines such as HL-60 and THP-1. Administration of HDAC inhibitors reduced the expression of Nox2 and Nox4 in monocrotaline-induced pulmonary hypertension [51]. The difference between previous investigations and our study is the animal model. A promising finding of our study is that MS-275 treatment attenuated all inflammatory markers including iNOS, TNFα, IL-1β, and MCP-1. MS-275 reduced adhesion molecules such as VCAM-1 and ICAM-1, as well as macrophage infiltration. A previous study reported similar results that valproate reduced inflammatory responses such as TNF, IL-1β, and IL-6 levels in the heart of SHR [40]. Various bodies of evidence have shown that HDAC inhibitors effectively suppress inflammation in animal models of inflammatory diseases such as arthritis, asthma, diabetes, and cardiovascular diseases [52, 53].

In conclusion, our results demonstrated that class I HDAC inhibitors reduced systolic blood pressure and arterial wall thickness in Ang II-induce mice through the downregulation of E2F3 and GATA6 expression. In addition, class I HDAC inhibitors induced vascular relaxation of the rat aortic rings through the NO signaling pathway, which needs to be investigated further in comprehensive studies. Interestingly, the HDAC inhibitor MS-275 exhibited anti-inflammatory effects on Ang II-induced hypertension. Thus, our results suggested that class I HDAC inhibitors have the potential for hypertension treatment.

## Supporting information

S1 Fig MS-275 reduces the mRNA level of collagen type III in angiotensin II-induced hypertensive mice. Collagen type III mRNA was estimated by qRT-PCR. **p < 0.01 versus sham group; ^###^p < 0.001 versus angiotensin II group; NS, not significant.
(TIF)

S2 Fig Negative control for CD68 macrophages in angiotensin II-induced hypertensive mice. Negative control showed no reactivity for CD68 in the angiotensin II aorta tissue. Scale bar = 50 μm.
(TIF)

## Abbreviations

ADMA: asymmetric dimethylarginine
ANP: atrial natriuretic peptide
BNP: brain natriuretic peptide
COX-2: cyclooxygenase-2
DDAH1: dimethylarginine dimethylaminohydrolase
GTPCH: guanosine-triphosphate (GTP) cyclohydrolase
ICAM-1: intercellular adhesion molecule-1
IL-1β: interleukin 1 beta
iNOS: inducible nitric oxide synthase
PRMT1: protein arginine methyltransferase 1
TNF-α: tumor necrosis factor alpha
PVDF: polyvinylidene difluoride
qRT-PCR: quantitative real-time reverse transcription polymerase chain reaction
SOD3: superoxide dismutase 3
VCAM-1: vascular cell adhesion molecule-1
VSMCs: vascular smooth muscle cells
