# Supplementary figures and images for: Class I histone deacetylase inhibitor MS-275 attenuates vasoconstriction and inflammation in angiotensin II-induced hypertension
Source: PLoS One. 2019 Mar 4;14(3):e0213186. doi: 10.1371/journal.pone.0213186 (PMC6398866; doi:10.1371/journal.pone.0213186)

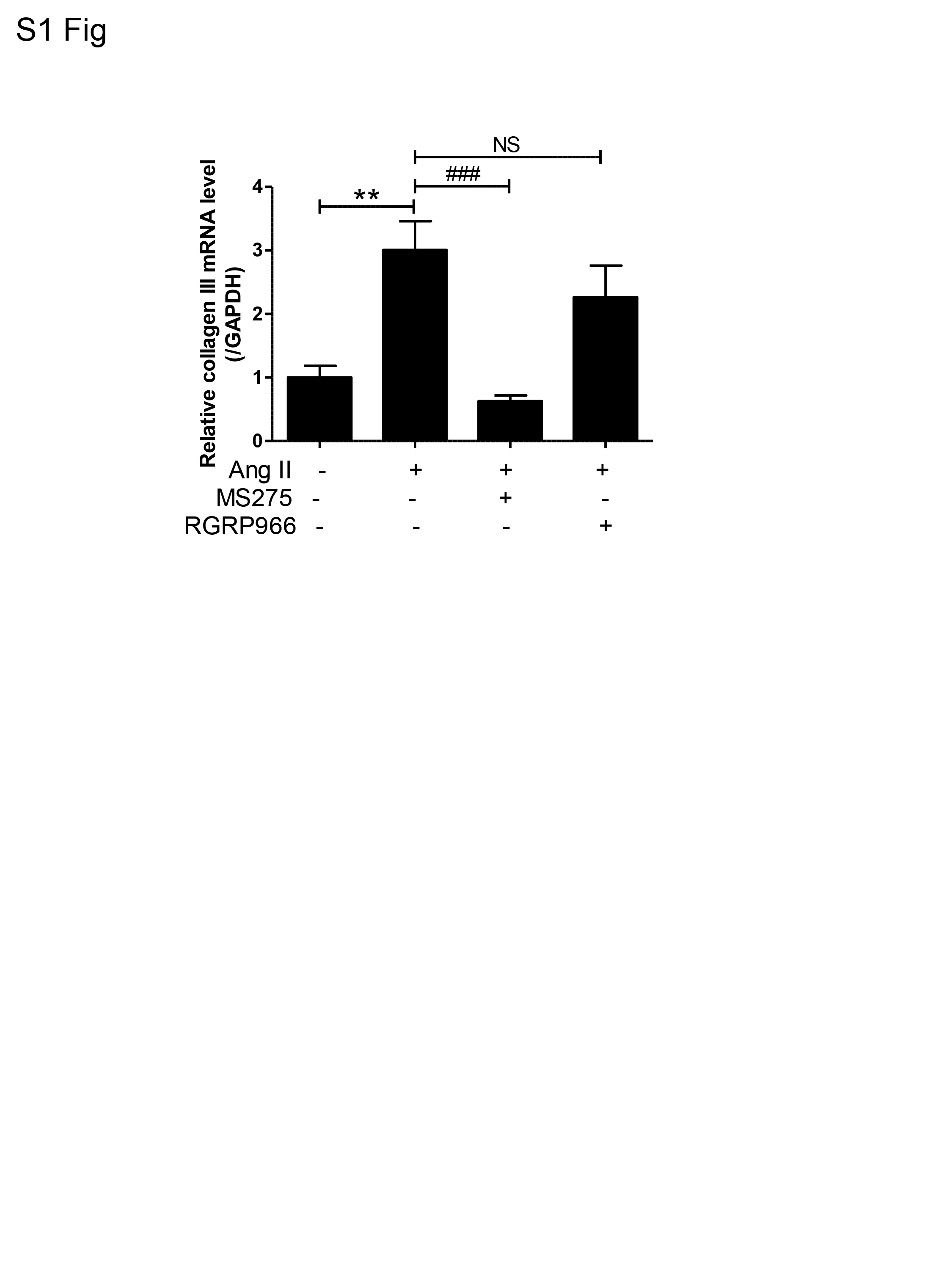

Supplement: S1 Fig — Collagen type III mRNA was estimated by qRT-PCR. **p < 0.01 versus sham group; ###p < 0.001 versus angiotensin II group; NS, not significant. (TIF) [file pone.0213186.s001.tif]

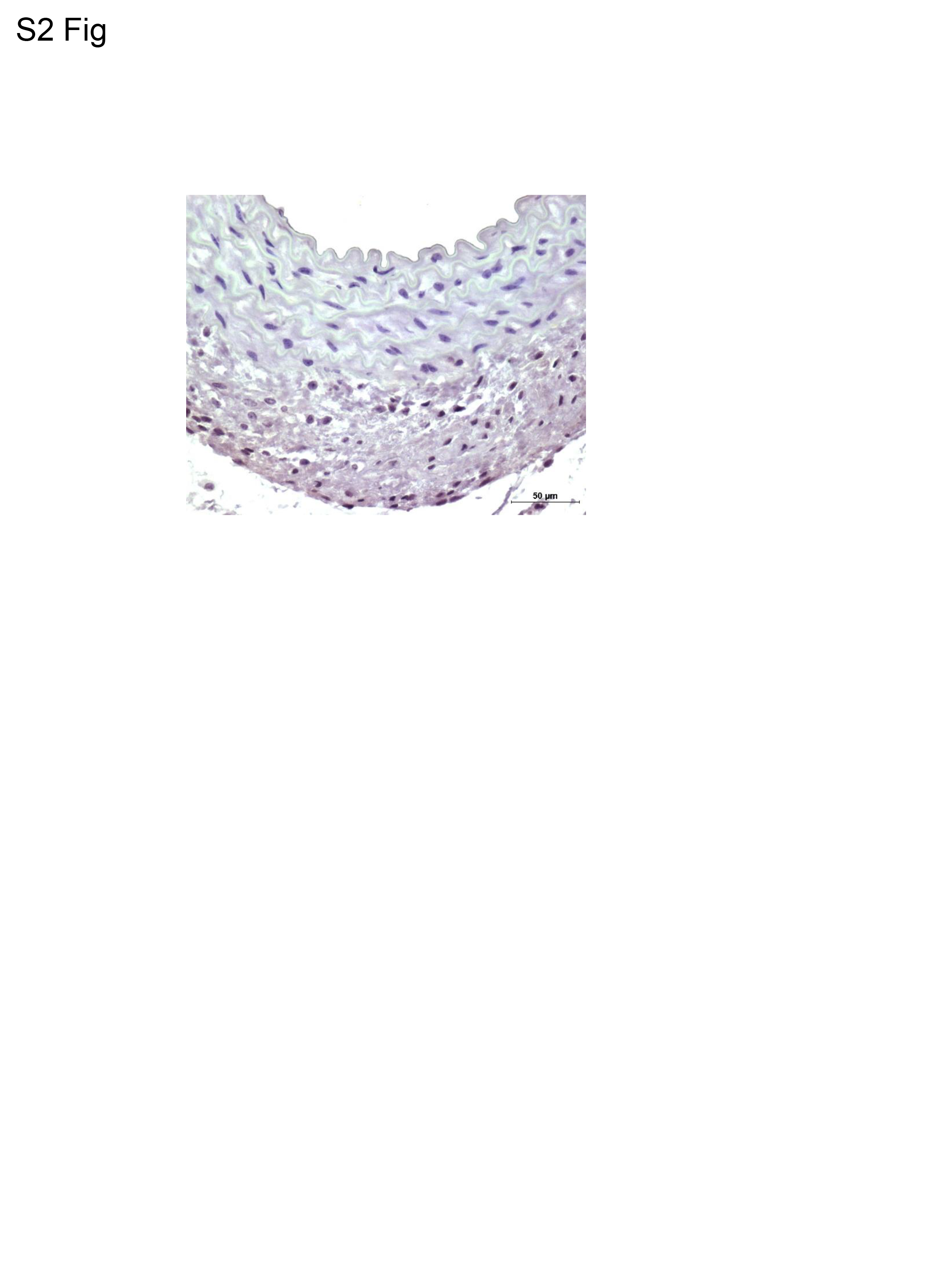

Supplement: S2 Fig — Negative control showed no reactivity for CD68 in the angiotensin II aorta tissue. Scale bar = 50 μm. (TIF) [file pone.0213186.s002.tif]
